# USP21 deubiquitylates Nanog to regulate protein stability and stem cell pluripotency

**DOI:** 10.1038/sigtrans.2016.24

**Published:** 2016-11-04

**Authors:** Xingyu Liu, Yuying Yao, Huiguo Ding, Chuanchun Han, Yuhan Chen, Yuan Zhang, Chanjuan Wang, Xin Zhang, Yiling Zhang, Yun Zhai, Ping Wang, Wenyi Wei, Jing Zhang, Lingqiang Zhang

**Affiliations:** 1 Department of Hepatology and Gastroenterology, Beijing Youan Hospital, Capital Medical University, Beijing, China; 2 State Key Laboratory of Proteomics, Beijing Proteome Research Center, Beijing Institute of Radiation Medicine, Collaborative Innovation Center for Cancer Medicine, Beijing, China; 3 Institute of Cancer Stem Cell, Dalian Medical University, Dalian, China; 4 College of Life Sciences, Xiamen University, Xiamen, China; 5 Department of Orthopedics, the General Hospital of Chinese People’s Liberation Army, Beijing, China; 6 Shanghai Key Laboratory of Regulatory Biology, Institute of Biomedical Sciences and School of Life Sciences, East China Normal University, Shanghai, China; 7 Department of Pathology, Beth Israel Deaconess Medical Center, Harvard Medical School, Boston, Massachusetts, USA; 8 Department of Microbiology and Immunology, Indiana University School of Medicine, Indianapolis, Indiana, USA

## Abstract

The homeobox transcription factor Nanog has a vital role in maintaining pluripotency and self-renewal of embryonic stem cells (ESCs). Stabilization of Nanog proteins is essential for ESCs. The ubiquitin–proteasome pathway mediated by E3 ubiquitin ligases and deubiquitylases is one of the key ways to regulate protein levels and functions. Although ubiquitylation of Nanog catalyzed by the ligase FBXW8 has been demonstrated, the deubiquitylase that maintains the protein levels of Nanog in ESCs yet to be defined. In this study, we identify the ubiquitin-specific peptidase 21 (USP21) as a deubiquitylase for Nanog, but not for Oct4 or Sox2. USP21 interacts with Nanog protein in ESCs *in vivo* and *in vitro*. The C-terminal USP domain of USP21 and the C-domain of Nanog are responsible for this interaction. USP21 deubiquitylates the K48-type linkage of the ubiquitin chain of Nanog, stabilizing Nanog. USP21-mediated Nanog stabilization is enhanced in mouse ESCs and this stabilization is required to maintain the pluripotential state of the ESCs. Depletion of USP21 in mouse ESCs leads to Nanog degradation and ESC differentiation. Overall, our results demonstrate that USP21 maintains the stemness of mouse ESCs through deubiquitylating and stabilizing Nanog.

## Introduction

Embryonic stem cells (ESCs) and induced pluripotent stem cells are pluripotent stem cell lines that have the ability to self-renew and differentiate into multiple lineages.^1^ The pluripotency of ESCs is controlled by a series of core transcription factors, such as Nanog, Oct4 and Sox2.^2–4^ Knowing the transcription factors that regulate the pluripotency of ESCs, human-induced pluripotent stem cells can be produced by the combined expression of these factors (that is, Oct4, Sox2, Nanog and LIN28 or Oct4, Sox2, c-Myc and KLF4).^5–7^ Nanog, first discovered in 2003 as a critical factor that underlies pluripotency in both the inner cell mass and ESCs, is capable of maintaining ESC self-renewal independent of leukemia inhibitory factor (LIF)/Stat3^4, 8^ and is expressed in pluripotent embryonic cells, derivative ESCs and the developing germ line of mammals and birds. *Nanog*-deficient inner cell mass failed to generate epiblast and only produced parietal endoderm-like cells. *Nanog*-deficient ESCs lost pluripotency and differentiated into extraembryonic endoderm lineage.^4,8^ Overexpression of Nanog allows mouse (m)ESCs to remain pluripotent even without LIF and enables human ESCs to grow feeder-free and form clones efficiently.^8,9^ In contrast, reduced expression of Nanog in ESCs leads to differentiation.^4,10^ So, it is not surprising that the tight regulation of Nanog expression levels is of great importance.

Previous studies have demonstrated that transcription factors are needed to activate and/or suppress Nanog expression. In this regard, Oct4 and Sox2 have been shown to be the main regulators of Nanog expression in mESCs.^11–13^ Oct4, Sox2 and Nanog often co-occupy a substantial portion of their target genes and collaborate to form regulatory circuitry consisting of autoregulatory and feed-forward loops. In addition, epigenetic factors, including Wdr5, Mof and Ezh2, can modulate Nanog transcription in ESCs.^14–16^

Recent studies showed that post-translational modifications (PTMs) also have important and complex roles in the control of pluripotency and cellular reprogramming. Ubiquitylation is one of the most important PTMs to control protein stability. The ubiquitin–proteasome system mediates the rapid and highly specific addition or removal of ubiquitin to intracellular proteins and thereby contributes to the dynamic regulation of protein abundance, including stem cell transcription factors. The E3 ubiquitin ligase, F-box and WD40 domain-containing protein 8 (FBXW8), has been recently reported to promote Nanog polyubiquitylation and degradation, and result in ESC differentiation.^17^ Interestingly, FBXW8 binding to Nanog requires the phosphorylation of Nanog at N-terminal Serines 52, 71 and 78 by the kinase ERK1. Consistent with this notion, a recent systemic ‘ubiproteome’ study in ESCs and induced pluripotent stem cells has found that the ubiquitin–proteasome system constitutes a major level of regulation of the pluripotency and reprogramming of these cells.^18^ More than 1000 proteins were identified to be ubiquitylated in mESCs in the pluripotent or differentiated states. Many well-characterized members of the extended pluripotency network, such as Nanog and Oct4, are themselves ubiquitylated in the pluripotent cells.^18^

Deubiquitylases (DUBs, also called deubiquitinases) can remove ubiquitin molecules from protein substrates and maintain their stability. There are ~90 DUBs in the human proteome including ubiquitin C-terminal hydrolyases (UCHs), ubiquitin-specific peptidases (USPs), ovarian tumor domain enzymes (OTUs), Josephins, Jad/Pad/MPN domain-containing metallo enzymes and monocyte chemotactic protein-induced proteins.^19–21^ So far, the DUB that regulates Nanog protein stability remains unknown.

In this study, we identified USP21 as an efficient DUB that reverses Nanog polyubiquitylation and stabilizes Nanog protein. Our study further demonstrates that depletion of USP21 in mESCs leads to Nanog degradation and mESC differentiation. These results provide a potential resource for cell substitutive therapy and drug discovery.

## Materials and methods

### Cell culture

Mouse ESC lines R1 and E14 were maintained in dishes without feeder cells but with 0.3% gelatin and α-minimum essential medium (Hyclone, Logan, UT, USA) supplemented with 10% fetal bovine serum (FBS; Hyclone), 1% penicillin–streptomycin solution, 2 mm l-glutamine (Invitrogen, Carlsbad, CA, USA), 100 μm non-essential amino acids (Invitrogen), 0.1 mm sodium pyruvate (Invitrogen), 0.1 mm β-mercaptoethanol (Sigma, St Louis, MO, USA) and 1000 U ml^−1^ LIF (Millipore, Temecula, CA, USA) at 37 °C and 5% CO_2_. For LIF withdrawal differentiation assays, mESCs were cultured in medium containing the supplements listed above at the concentrations indicated but without LIF. Human embryonic carcinoma NCCIT cells and human embryonic kidney HEK293T cells were cultured with Dulbcco's modified Eagle's medium supplemented with 10% fetal bovine serum.

### Plasmids and antibodies

Human DUB library was purchased from OriGene (Rockville, MD, USA). USP21 and Nanog full-length and mutants were sub-cloned into pCMV-Myc or pFlag-CMV-2 vectors as indicated. GST-tagged USP21 and Nanog were inserted into the pGEX-6P-1 or pET-28a vector. Cells were transfected with various plasmids using TuboFect reagent (Thermo, Waltham, MA, USA) according to the manufacturer’s protocol. The antibodies we used in this study are as follows: anti-Flag (MBL, Woburn, MA, USA), anti-Myc (MBL), anti-Nanog (Bethyl, Montgomery, UK; CST), anti-USP21 (Santa Cruz Inc., Santa Cruz, CA, USA), anti-Oct4 (Santa Cruz Inc.), anti-His (Santa Cruz Inc.), anti-GST (Santa Cruz Inc.), anti-HA (MBL), anti-actin (Santa Cruz Inc.), anti-mouse secondary antibody (Sigma), anti-rabbit secondary antibody (Sigma) and anti-goat secondary antibody (Abbkine, Redlands, CA, USA).

### Lentiviral packaging for knockdown experiment

USP21 was knocked down in ESCs and NCCIT cells using a lentiviral system. Short hairpin RNAs (shRNAs) were constructed by inserting shRNA sequences into a pGIPZ vector. The sense sequences of shRNA were as following: #1: 5′-TGCTAGAAGAACCTGAGTT-3′, #2: 5′-CAGTTGAAAAGTTGTCTCA-3′, #3: 5′-TCACTAAGGAAGAAGAGCT-3′ and #4: 5′-AACCTAATGTGGAAACGTT-3′. Infective lentiviruses were produced by co-transfection of the expression vector and packaging plasmids into 293FT cells, which were added to NCCIT cells and ESCs.

### Co-immunoprecipitation assay

Cells were lysed in HEPES buffer (20 mm HEPES, 50 mm NaCl, 0.5% Triton-X 100, 1 mm NaF) supplemented with protease inhibitor cocktail and dithiothreitol. The cell lysates were used for co-immunoprecipitation assays. Cell lysates were immunoprecipitated with the indicated antibodies for 3 h at 4 °C and then incubated with protein A/G (Santa Cruz Inc.) for overnight at 4 °C. Protien A/G-agarose was washed with HEPES buffer three times and then analyzed by western blot with the indicated antibodies.

### GST pull-down assay

*Escherichia coli* BL21-expressing GST or GST-USP21 proteins were immobilized on glutathione-Sepharose 4B beads then washed. These beads were next incubated with His-Nanog that was expressed in BL21 and purified by Ni-sepharose-agarose beads for 8–12 h at 4 °C. Then, the beads were washed with elution buffer and then proteins were eluted for western blotting.

### *In vivo* ubiquitylation assay

For Nanog ubiquitylation analysis, HEK293T cells were transfected with HA-ubiquitin, Myc-USP21, Myc-USP21CA or Flag-Nanog as indicated. Cells were treated with the proteasome inhibitor MG132 (20 μm; Sigma) for 8–10 h. At 36 h after transfection, cells were lysed in RIPA buffer (50 mm Tris-HCl, 1% NP-40, 1% sodium deoxycholate, 10% glycerinum, 150 mm NaCl, 5 mm EDTA, 0.1% SDS) and then incubated with anti-Flag antibody for 3 h and protein A/G-agarose beads overnight at 4 °C. After washing three times, ubiquitylated Nanog was detected by immunoblotting using anti-HA monoclonal antibody.

### *In vitro* deubiquitylation assay

Flag-Nanog and HA-ubiquitin were co-expressed in HEK293T cells. After treatment with the proteasome inhibitor MG132 (10 μm) for 8 h, the ubiquitylated proteins were purified by immunoprecipitation with anti-Flag antibodies. GST-USP21 protein purified from *E. coli* and the ubiquitylated Nanog was incubated in elution buffer for 30 min at 25 °C. The samples were then resolved by SDS-polyacrylamide gel electrophoresis followed by immunoblot analysis using anti-HA antibody.

### RNA extraction and real-time RT-PCR

Total cell RNA was prepared using Trizol reagent (Sigma) following the manufacturer’s instructions. First strand complementary DNA was synthesized using ReverTra Ace qPCR RT Master Mix kit (TOYABO, Osaka, Japan) following the manufacturer’s instructions. Real-time quantitative PCR was performed using a KAPA SYBR FAST qPCR kit (Kapa Biosystems, Wilmington, MA, USA). The sequences of real-time PCR primers are below. GAPDH-RT-forward (F): 5′-TGTGTCCGTCGTGGATCTGA-3′, GAPDH-RT-Reverse (R): 5′-CACCACCTTCTTGATGTCATCATAC-3′; Nanog-RT-F: 5′-CTCATCAATGCCTGCAGTTTTTCA-3′, Nanog-RT-R: 5′-CTCCTCAGGGCCCTTGTCAGC-3′; Rex1-RT-F: 5′-ACGAGGTGAGTTTTCCGAAC-3′, Rex1-RT- R: 5′-CCTCTGTCTTCTCTTGCTTC-3′; Oct4-RT-F: 5′-TCTTTCCACCAGGCCCCCGGCTC-3′, Oct4-RT-R: 5′-TGCGGGCGGACATGGGGAGATCC-3′; Sox2-RT-F: 5′-TAGAGCTAGACTCCGGGCGATGA-3′, Sox2-RT-R: 5′-TTGCCTTAAACAAGACCACGAAA-3′; Gata4-RT-T: 5′-TGGAAGACACCCCAATCTCG-3′, Gata4-RT-R: 5′-TAGTGTCCCGTCCCATCTCG-3′; Nestin-RT-F: 5′-CT GCAGGCCACTGAAAAGTT-3′, Nestin-RT-R: 5′-GACCCTGCTTCTCCTGCTC-3′; USP21-RT-F: 5′-GCAGGATGCCCAAGAGTT-3′, USP21-RT-R: 5′-GCAGGGACAGGTCACA AAA-3′.

### Cytoplasmic and nuclear fractionation

R1 cells were collected and washed with ice-cold phosphate-buffered saline twice. Cells were lysed in 250 μl lysis buffer (10 mm HEPES-NaOH (pH 7.9), 10 mm KCl, 1.5 mm MgCl_2_, 0.5 mm β-mercaptoethanol) supplemented with protease inhibitor mixture and phosphatase inhibitor for 15 min then lysis buffer plus 10% NP-40 was added for another 2 min. The lysate was then centrifuged at 16 000 *g* for 10–15 min. After collecting the supernatant containing the cytoplasmic fraction, the pellet was further lysed in nuclear lysis buffer (10 mm Tris-HCl (pH 7.6), 420 mm NaCl, 0.5% Nonidet P-40, 2 mm MgCl_2_, 1 mm dithiothreitol, 1 mm PMSF and 1% protease inhibitor cocktail) for 20 min. After centrifugation, the supernatant, constituting the nuclear fraction, was collected for further analysis.

### Protein half-life assay

For Nanog protein half-life assays, cellular transfection was performed when cells cultured in 2 cm plates reached ~60% confluence. Twenty-four hours later, cells were treated with the protein synthesis inhibitor cycloheximide (Sigma, 10 μg ml^−1^) for the indicated durations before harvest.

### Alkaline phosphatase staining

Alkaline phosphatase staining was carried out using the Leukocyte Alkaline Phosphatase kit (Sigma). Cells were washed twice with phosphate-buffered saline and fixed with fixative solution for 30 s at room temperature. The cells were rinsed gently in deionized water twice and added to a alkaline-dye mixture and then incubated at room temperature for 30 min followed by being washed with deionized water. Alkaline phosphatase-positive colonies were observed under a light microscope (Olympus, Tokyo, Japan).

### Statistics analysis

Statistical comparisons between two groups were carried out by Student’s *t*-test and one-way analysis of variance. A two-tailed *P*-value <0.05 was considered significant.

## Results

### DUB USP21 stabilizes Nanog protein

**Figure 1:**
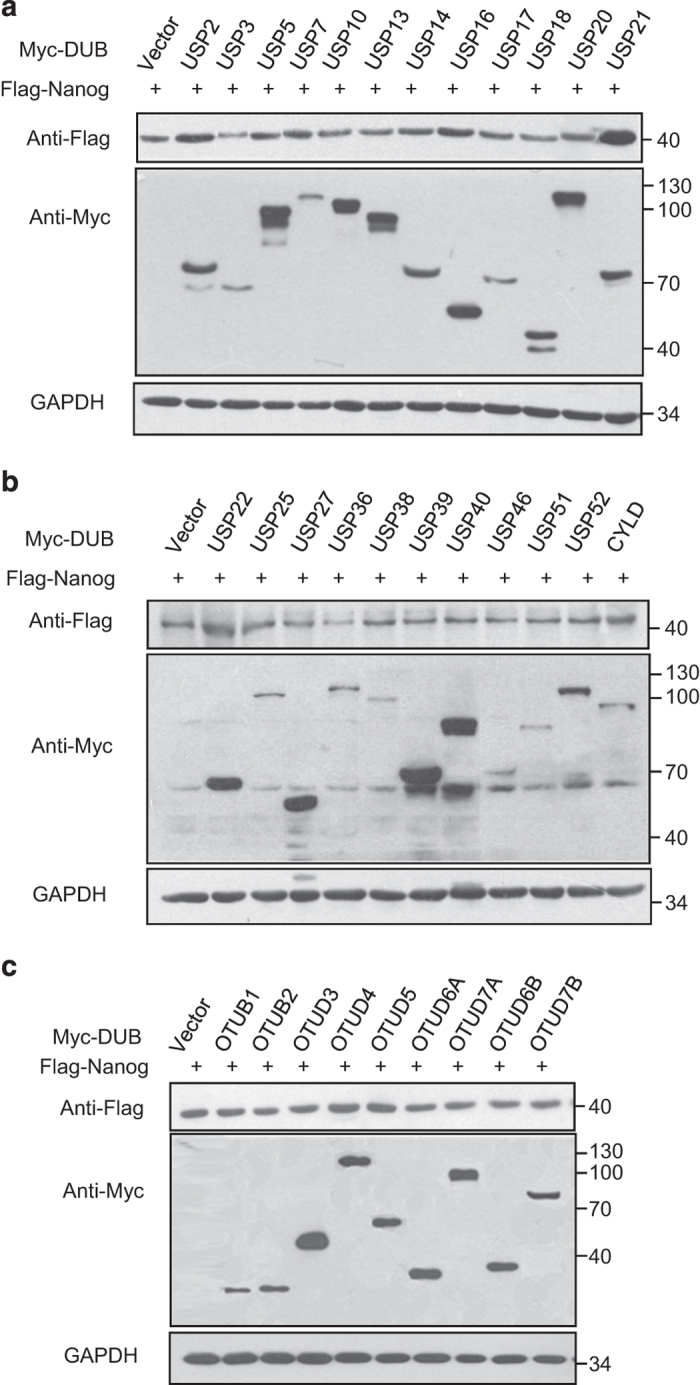
The deubiquitylase screen identifies USP21 as a candidate for Nanog regulation. (**a**, **b**) Flag-Nanog and the indicated USP subfamily DUBs were co-transfected into HEK293T cells. Forty-eight hours later, cell lysates were subjected to western blot. (**c**) Flag-Nanog and the indicated OTU subfamily DUBs were co-transfected into HEK293T cells. Forty-eight hours later, cell lysates were subjected to western blot.

**Figure 2:**
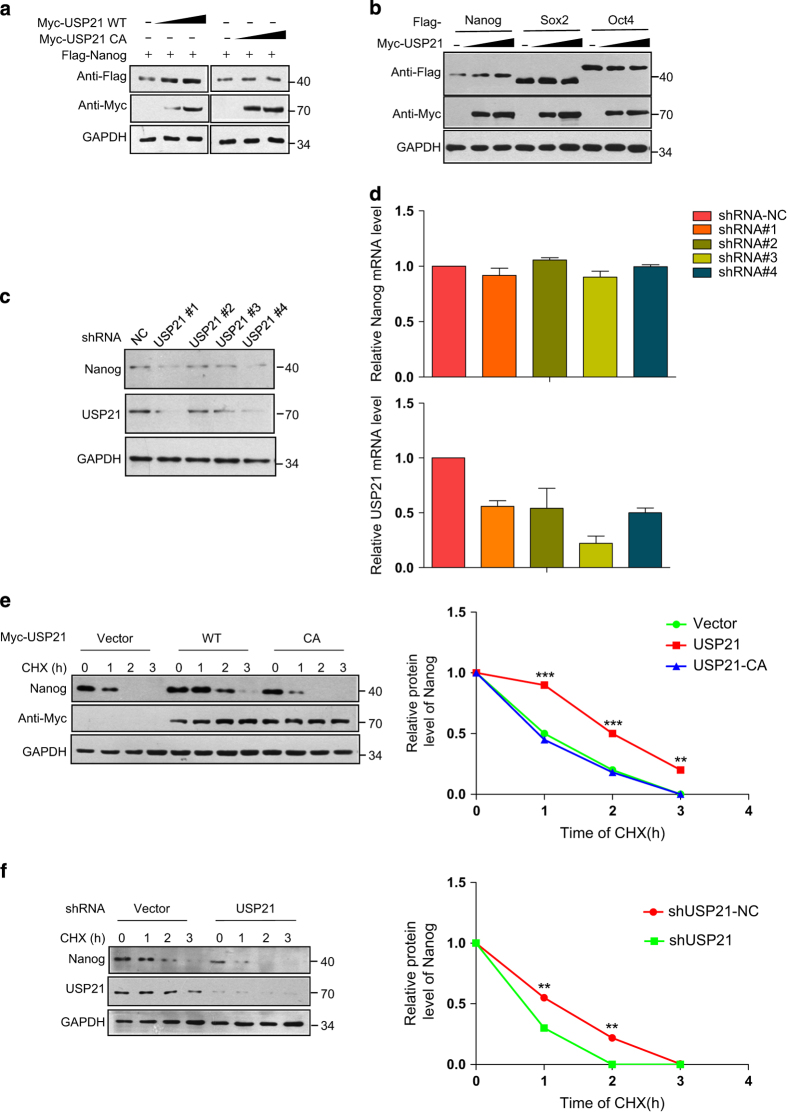
The deubiquitylase USP21 stabilizes Nanog protein in stem cells. (**a**) Flag-Nanog was co-transfected with increasing amounts of USP21 WT or C221A mutant into HEK293T cells. Forty-eight hours later, Nanog expression was detected. (**b**) Nanog, Sox2 or Oct4 were each co-transfected with increasing amounts of USP21 construct DNA into HEK293T cells. Forty-eight hours later, Nanog, Sox2 or Oct4 expression were detected. (**c**) Nanog was analysed in NCCIT cells transfected with individual USP21 shRNA. (**d**) Depletion of USP21 had no statistically significant effect on Nanog messenger RNA (mRNA) level in NCCIT cells. *n*=3 independent experiments. (**e**) NCCIT cells transfected with the control vector, Myc-USP21 WT or Myc-USP21-C221A were treated with cycloheximide (CHX, 10 μg ml^−1^) for the indicated time intervals, and protein levels of endogenous Nanog and ectopic USP21 were detected by western blotting. Quantification of Nanog levels relative to GAPDH is shown on the right. Data are the representative results of three independent experiments. **P<0.01, ***P<0.001, Student’s *t*-test. (**f**) NCCIT cells transfected with the control shRNA or USP21 shRNA were treated with CHX for the indicated times, and protein levels of Nanog, USP21 were detected by western blotting. Quantification of Nanog levels relative to GAPDH is shown on the right. **P<0.01 Student’s *t*-test.

To identify the potential DUB for Nanog, a screen examining a total of 32 DUBs, including USP and OTU subfamilies, was performed. Given that endogenous Nanog is lowly expressed in the human embryonic kidney cell line HEK293T, we overexpressed the ectopic DUB and Nanog in the cells and analysed the expression of Nanog. Through the screen, we found that USP21 significantly upregulated Nanog levels, whereas other DUBs had little to no effect on the Nanog expression levels (Figures 1a–c). Overexpression of USP21, but not the catalytically inactive mutant C221A, led to increased Nanog levels in a dose-dependent way (Figure 2a), suggesting that USP21 upregulation of Nanog expression is dependent on DUB enzyme activity. The effect of USP21 on Nanog is specific since overexpression of USP21 seemed to have little effect on Sox2 and Oct4 (Figure 2b), two well-characterized transcription factors in stem cells. It has been demonstrated that Nanog is abnormally overexpressed in human embryonic carcinoma NCCIT cells, which demonstrate similar gene expression profiles as ESCs.^22^ We then knocked down the endogenous USP21 in NCCIT cells and examined the effects on endogenous Nanog. Depletion of endogenous USP21 expression with a shRNA appreciably downregulated Nanog protein in the NCCIT cells and similar effects were observed with four individual shRNAs (Figure 2c). However, the messenger RNA levels of Nanog in NCCIT cells were not remarkably affected by depletion of USP21 (Figure 2d). These results suggest that USP21 has a role in the maintenance of Nanog abundance.

To further confirm that USP21 regulates the stability of Nanog, we examined endogenous Nanog protein levels in the NCCIT cells treated with cycloheximide, a protein synthesis inhibitor. The half-life of endogenous Nanog protein in NCCIT cells is ~1 h. Overexpression of USP21 prolonged the protein half-life of Nanog to ~2 h, whereas the catalytically inactive mutant USP21 C221A had no such effects (Figure 2e). Conversely, depletion of endogenous USP21 with shRNA shortened the protein half-life of Nanog (Figure 2f). These results indicate that USP21 specifically stabilizes Nanog protein, with its effect is dependent on the DUB activity of USP21.

### USP21 interacts with Nanog *in vivo* and *in vitro*

**Figure 3:**
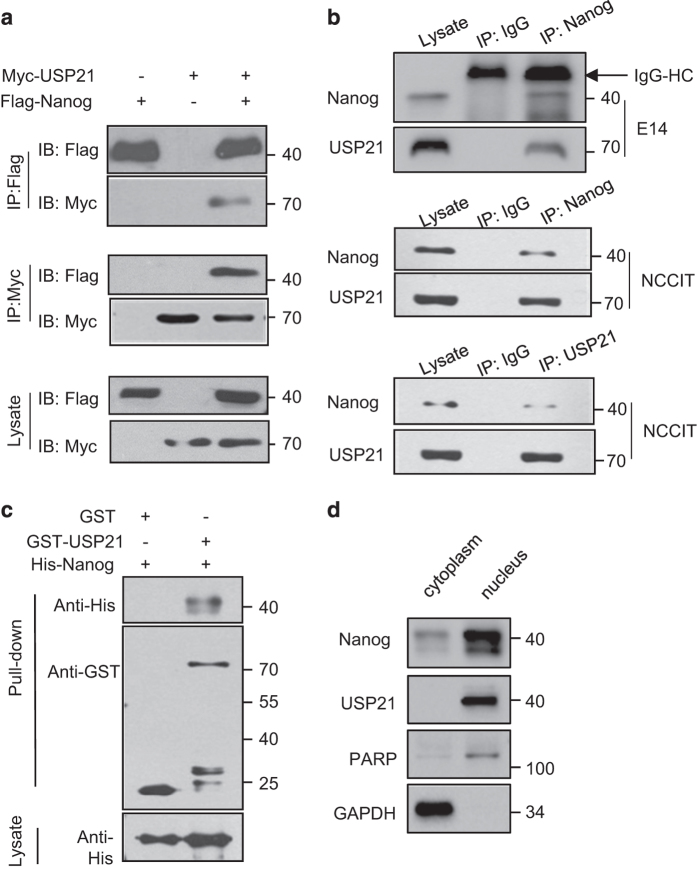
USP21 interacts with Nanog both *in vivo* and *in vitro*. (**a**) HEK293T cells co-transfected with Flag-Nanog and Myc-USP21 were subjected to immunoprecipitation (IP) with anti-Flag antibodies or anti-Myc antibodies. The immunoprecipitates and lysates were analysed by western blotting. (**b**) E14 and NCCIT cells were subjected to immunoprecipitation (IP) with control IgG, anti-Nanog or USP21 antibodies. The immunoprecipitates were detected by western blotting. (**c**) GST and GST-USP21 proteins purified from *E. coli* were incubated with His-Nanog protein. Proteins retained on Sepharose were blotted with the anti-His or anti-GST antibody. (**d**) Cytoplasmic and nuclear fractions of NCCIT cells were separated by cytoplasmic and nuclear fractionation. Western blot assay was then performed. PARP and GAPDH represent the nuclear and cytoplasmic marker protein, respectively.

To investigate whether USP21 interacts with Nanog, co-immunoprecipitation assays were performed. We found that ectopic Nanog and USP21 were reciprocally co-immunoprecitated, and that their interaction was independent of the DUB activity of USP21 (Figure 3a). Importantly, endogenous Nanog physiologically interacted with endogenous USP21 in mESCs (E14) and human embryonic carcinoma NCCIT cells (Figure 3b). To investigate whether the interaction between Nanog and USP21 was direct, we then performed GST pull-down assays. The results showed that purified GST-USP21 but not GST alone could bind to His-Nanog *in vitro* (Figure 3c), indicating a direct interaction between Nanog and USP21. To evaluate the subcellular localization of Nanog and USP21, nuclear/cytoplasmic fractionation was performed. Once the cytoplasmic and nuclear fractions of the mouse ESC R1 cells were separated, we found that Nanog and USP21 were both predominantly detected in the nucleus of stem cells (Figure 3d).

**Figure 4:**
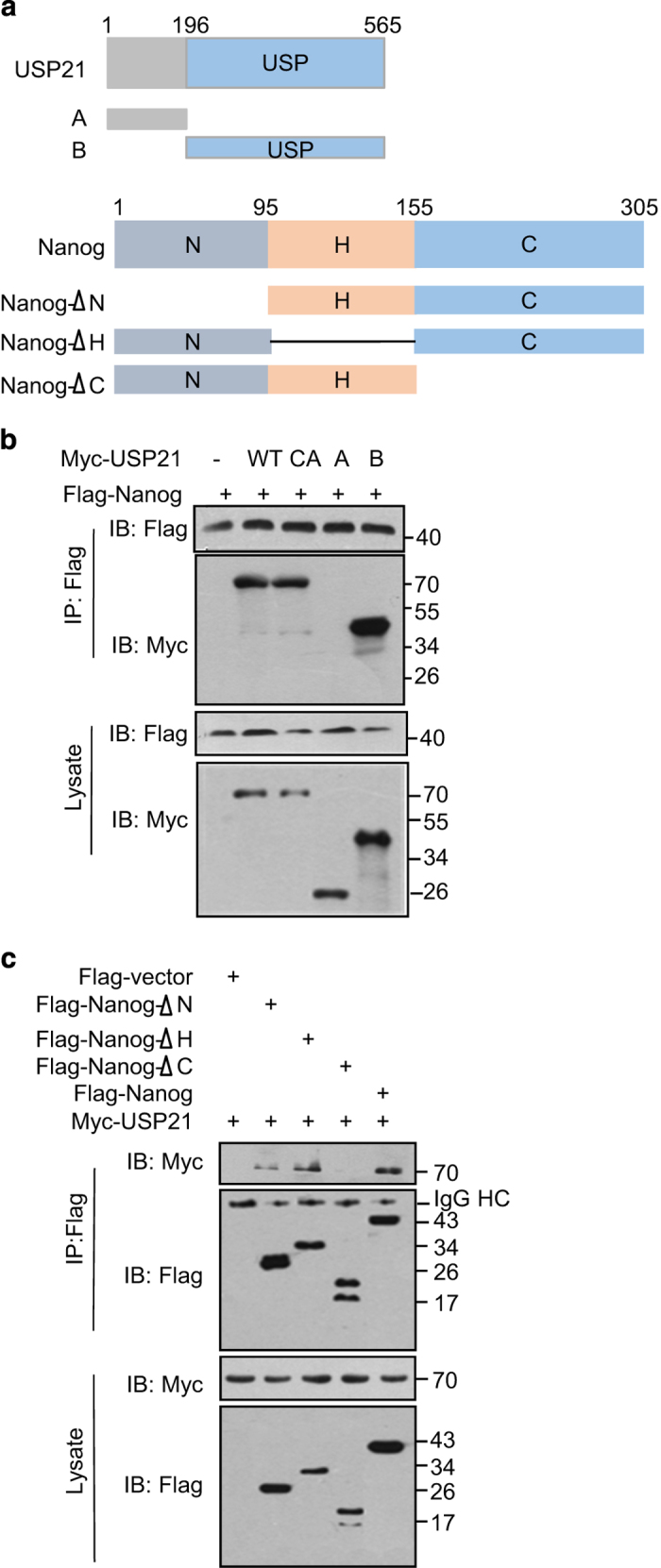
Mapping the binding regions between USP21 and Nanog proteins. (**a**) Overview of USP21 and Nanog structures. (**b**) HEK293T cells transfected with Flag-Nanog and the indicated Myc-USP21 deletion mutants were subject to immunoprecipitation with anti-Flag antibodies. The lysates and immunoprecipitates were blotted with anti-Flag and anti-Myc antibodies. (**c**) HEK293T cells transfected with Myc-USP21 and the indicated Flag-Nanog deletion mutants were subject to immunoprecipitation with anti-Flag antibodies. The lysates and immunoprecipitates were blotted with anti-Flag and anti-Myc antibodies.

To map the binding region mediating the interaction between Nanog and USP21, a series of deletion mutants were constructed (Figure 4a). Co-immunoprecipitation assays showed that the C-terminal USP domain of USP21 mediated its interaction with Nanog (Figure 4b). The C-domain of Nanog, but not the N domain nor the H (homeobox) domain of Nanog, was required for its interaction with USP21 (Figure 4c). Taken together, the results indicate that USP21 can interact with Nanog both *in vivo* and *in vitro*.

### USP21 deubiquitylates Nanog

**Figure 5:**
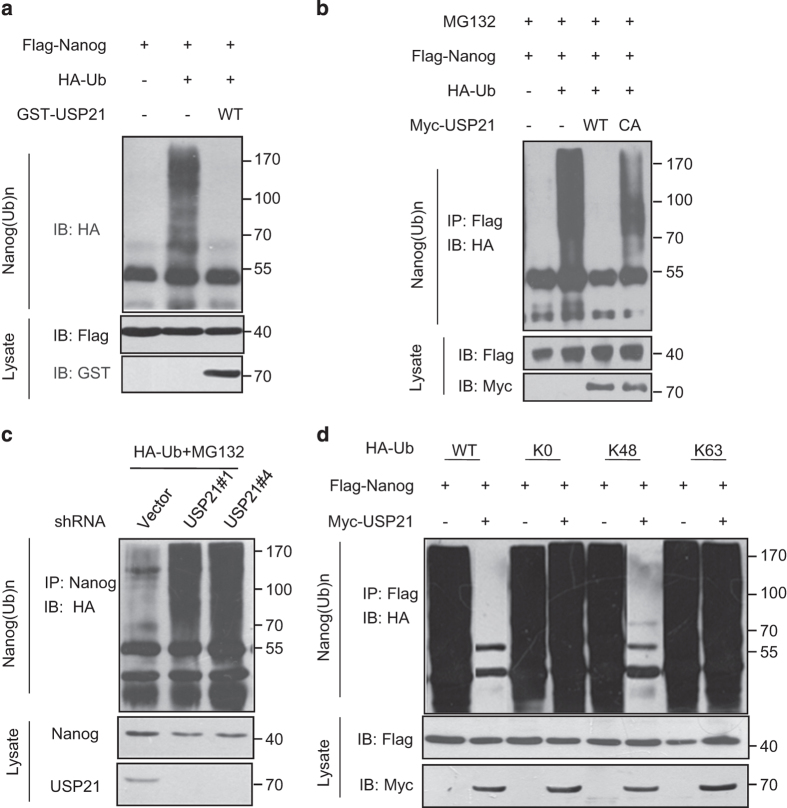
USP21 deubiquitylates Nanog and efficiently removes the K48-linkage type of polyubiquitin chain. (**a**) Flag-Nanog and HA-ubiquitin were co-expressed in HEK293T cells. After MG132 (10 μm) treatment for 8 h, the ubiquitylated proteins was purified by immunoprecipitation with anti-Flag antibodies. GST-USP21 protein purified from *E. coli* and the ubiquitylated Nanog were mixed and incubated in elution buffer. Then, the mixtures were analyzed via western blotting. (**b**) HEK293T cells transfected with the indicated constructs were treated with MG132 for 8 h before collection. The whole-cell lysate was subjected to immunoprecipitate with anti-Flag and western blot with anti-HA. (**c**) NCCIT cells transfected with the indicated shRNA were treated with MG132 for 8 h before collection. Nanog was immunoprecipitated with anti-Nanog and immunoblotted with anti-HA. (**d**) The Nanog ubiquitylation linkage was analysed in HEK293T cells transfected with Nanog, USP21 and the indicated ubiquitin Lys 0, Lys 48-only or Lys 63-only plasmids.

As USP21 is a DUB, we hypothesized that USP21 stabilizes Nanog through deubiquitylating Nanog. Indeed, we showed that USP21 reduced Nanog ubiquitylation directly in an *in vitro* deubiquitylation assay (Figure 5a). Ectopic expression of wild-type USP21, but not the C221A mutant of USP21, removed the ubiquitin chain of Nanog in cultured cells (Figure 5b). Consistent with this notion, downregulation of USP21 by two individual shRNAs increased Nanog ubiquitylation in NCCIT cells (Figure 5c). Notably, USP21 efficiently removed K48-linked polyubiquitylation of Nanog, but had no significant effect on monoubiquitylation or the nondegradative K63-linked polyubiquitylation of Nanog (Figure 5d). Collectively, these data suggest that USP21 can deubiquitylate and stabilize Nanog.

### Knockdown of USP21 induces mouse ESC differentiation

**Figure 6:**
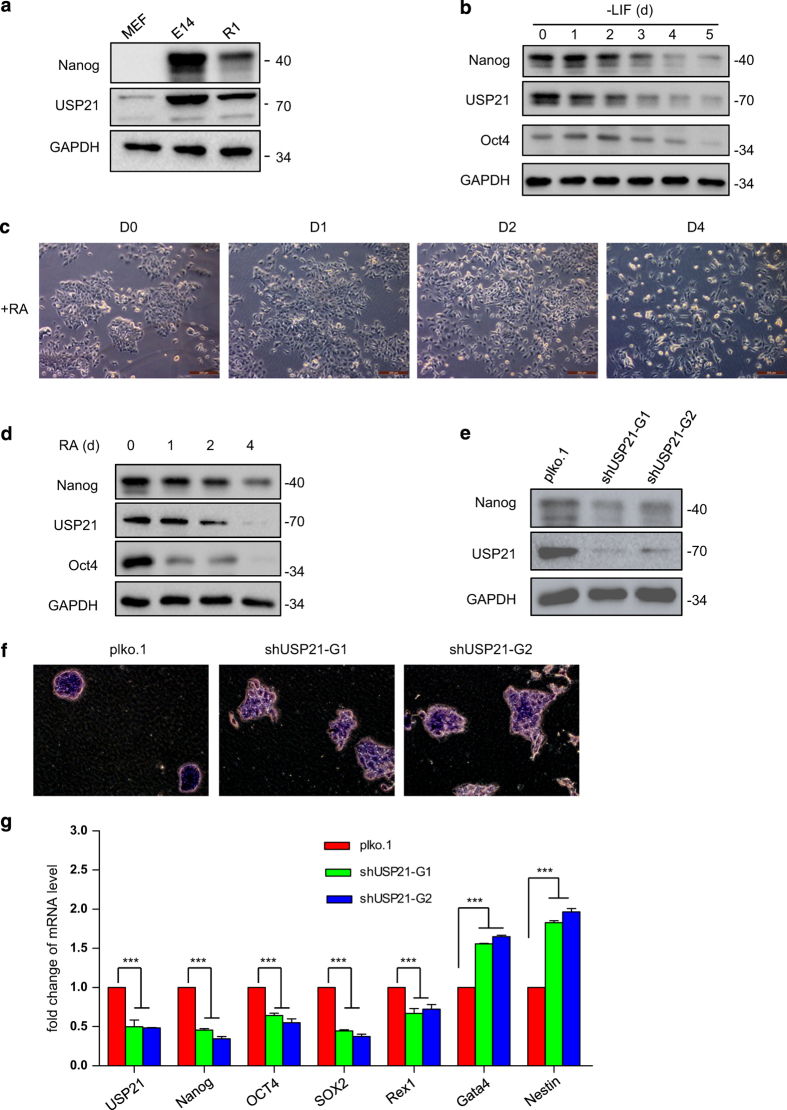
Knockdown of USP21 induces mouse ES cell differentiation. (**a**) Western blotting analysed the endogenous Nanog protein expression in mouse embryonic fibroblast (MEF), R1 and E14 cells. (**b**) R1 cells were collected after being cultured in the LIF withdrawal culture medium for the indicated time points. Expression of USP21, Nanog and Oct4 was tested by western blotting. (**c**, **d**) After treatment with 0.5 μm retinoic acid (RA) for 0, 1, 2 and 4 days, the morphology of R1 cells was examined under a light microscope and the protein levels of Nanog, Oct4, USP21 and GAPDH were detected by western blot with the indicated antibodies. (**e**–**g**) USP21 was knocked down by lentiviral approach in R1 cells. The expression levels of USP21, Nanog and GAPDH were analyzed with the indicated antibodies (**e**). Cell morphology was examined under a light microscope and the cells were stained by alkaline phosphatase (AP) to indicate the stemness (**f**). In addition, the expression of stemness markers and differentiation markers was analyzed by real-time RT-PCR (**g**). Data in **g** are shown as the mean±s.d. (*n*=3). ***P<0.001, Student’s *t*-test.

Nanog is essential to maintain the stemness of ESCs. Considering that USP21 deubiquitylates Nanog, we sought to examine the functional roles of USP21 in mESCs. First of all, we examined the protein levels of USP21 and Nanog in mouse embryonic fibroblast cells and mESCs E14 and R1. The protein expression of USP21 and Nanog in mouse embryonic fibroblast was much lower than that in E14 and R1 cells (Figure 6a), suggesting that USP21 is highly expressed in undifferentiated cells. After removal of LIF, the mESCs tended to differentiate and the expression of USP21 gradually decreased together with the pluripotency factors Oct4 and Nanog (Figure 6b). To verify these findings, we tested an additional differentiation induction model, which was induced by retinoic acid. During the induced differentiation, the morphology of ESCs significantly changed (Figure 6c). Meanwhile, the protein levels of USP21, Nanog and Oct4 were simultaneously reduced (Figure 6d). These data suggest that higher level of USP21 may be related to the pluripotency of ESCs.

To further examine the function of USP21 in mESCs, we knocked down USP21 in mESC R1 cells. This led to decreased Nanog expression (Figure 6e) and morphological changes in mESC that correlates with decreased alkaline phosphatase staining (Figure 6f). Consistently, after USP21 was knocked down, the messenger RNA levels of pluripotency markers decreased, while that of differential markers increased (Figure 6g). These results indicate that USP21 has a role in maintaining ESCs in an undifferentiated state.

## Discussion

Stem cell transcription factors are highly regulated at the levels of messenger RNA stability, translation and protein stability by PTMs.^22^ Among the PTMs, ubiquitylation is a major regulator of stem cell transcription factors, which can directly affect the pluripotency of stem cells. Previous studies have found that several core stem cell transcription factors undergo ubiquitylation and proteasomal degradation. For instance, the stability and transcriptional activity of Oct4 is regulated by Itch, a C2-WW-HECT domain type of ubiquitin E3 ligase. Oct4 degradation impairs ESC self-renewal and pluripotency.^23^ WWP2, another C2-WW-HECT type of E3 ligase, can interact with both Oct4 and Sox2, resulting in Oct4 and Sox2 downregulation and ESC differentiation.^24,25^ In addition to the HECT-type E3 ligases, the Cullin–RING type of E3s also have a critical role in regulation of stem cell transcription factors. In this regard, E3 ligases SCF^β-TrCP1^ and SCF^β-TrCP2^ have been shown to degrade Klf4,^26,27^ whereas SCF^Skp2^ and SCF^Fbw7^ are responsible for rapid c-Myc degradation.^28–30^ Nanog can maintain the stemness of ESCs and reprogram somatic cells; however, the PTMs of Nanog are still poorly understood. It has been reported that Nanog is phosphorylated at multiple sites by protein kinase C, focal adhesion kinase, ERKs and CDK1.^17,31–34^ Recently, the ubiquitin ligase for Nanog has been identified, showing that the F-box protein FBXW8 can reduce Nanog protein stability.^17^ ERK1, but not ERK2, phosphorylates Nanog on Serines 52, 71 and 78, and this phosphorylation induces the binding of FBXW8 with Nanog to reduce Nanog protein stability. A previous study showed that the PEST (enriched in Pro, Glu, Ser and Thr residues) motif in the N-terminal part of Nanog (amino acids 47–72) is involved in the rapid proteolysis and ubiquitylation of Nanog.^35^ Two phosphorylation sites of Nanog by ERK1 are located within the PEST motif (that is, Ser 52 and Ser 71). However, whether FBXW8 promotes the ubiquitylation of Nanog through forming a Cullin–RING complex (that is, FBXW8 acts as the substrate recognition subunit of the E3 complex, similar to the pattern of SCF^Fbw7^ and SCF^β-TrCP^) or not remains undefined, which is worthy of further investigations.

All the major PTMs, including the ubiquitylation process, can be reversed. Ubiquitylation and deubiquitylation work properly in a balanced manner to maintain stemness and permit differentiation.^20^ Although several ubiquitin ligases of core stem cell transcription factors have been shown, reports on the functions of DUBs are limited. Recent evidence has revealed that c-Myc can be deubiquitylated and regulated by USP28, USP36 and USP37.^36–38^ Oct4 protein expression is decreased by depletion of Psmd14, a deubiquitylating enzyme that resides in the 19S ‘lid’ of the proteasome. In addition, Psmd14 expression is essential for generating induced pluripotent stem cells.^18^ However, the mechanism of Sox2, Oct4 and Nanog protein regulation by DUBs is not well understood. In this study, we identified USP21 as a candidate DUB for Nanog, but not Sox2 nor Oct4, and showed that USP21 sustains the stemness of mESCs by stabilization of Nanog. It is notable that USP21 expression is decreased during the ESC differentiation, in contrast to the E3 ligase FBXW8, which remains unchanged during this process. So far as we know, this is the first time a specific DUB for Nanog has been identified.

**Figure 7:**
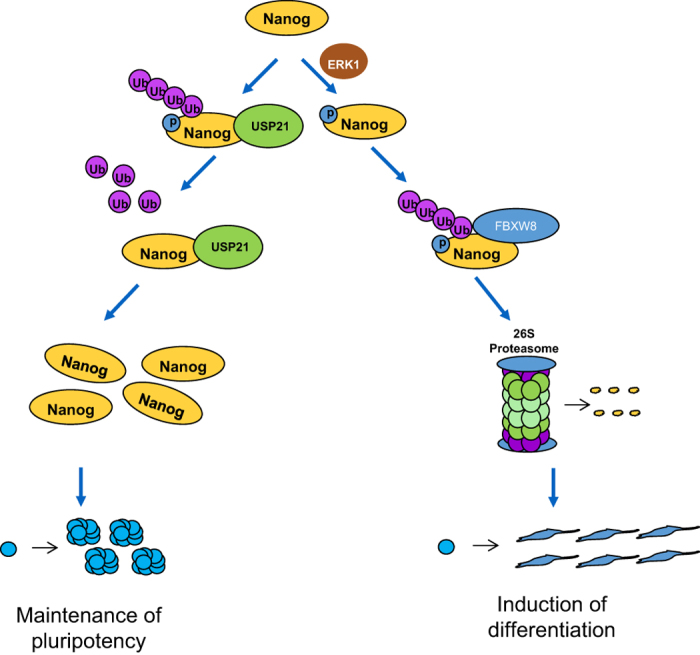
Control model of Nanog protein by DUB and E3 at the post-translational level. In the embryonic stem cells, the deubiquitylase USP21 is highly expressed, and USP21 deubiquitylates and stabilizes Nanog protein to promote ESC maintenance. In contrast, during the differentiation, USP21 expression declines, and the activated ERK1 phosphorylates Nanog and promotes the E3 ligase FBXW8-mediated ubiquitylation of Nanog. The net balance between the ubiquitylation and deubiquitylation controls Nanog protein levels and determines cellular fate.

Our current findings, together with previous reports, suggest a model in ESCs in which DUBs, such as USP21, are highly expressed and help maintain high levels of Nanog, supporting ESC stemness and pluripotency (Figure 7). During development, USP21 expression declines (although the molecular mechanism remains unclear and needs further investigation), whereas activated ERK1 phosphorylates Nanog and promotes the E3 ligase FBXW8-mediated ubiquitylation of Nanog. The latter event leads to the degradation of Nanog and induction of differentiation (Figure 7). Therefore, USP21, FBXW8 and ERK1 cooperate with each other to precisely regulate the ‘Yin-Yang’ balance of the stability and activity of Nanog under the undifferentiated and differentiated states of ESCs. These findings highlight the role of the network of protein ubiquitylation and phosphorylation in stem cell control.

Among the whole family of DUBs, consisting of ~100 enzymes, USPs are the largest subfamily, which contain >50 members and include conserved domains and catalytic sites.^19,39,40^ In this study, we screened the USP and OTU subfamilies and identified USP21 as a DUB for Nanog. However, we cannot rule out the possibility that additional DUBs belonging to the other subfamilies such as ubiquitin C-terminal hydrolyase, Jad/Pad/MPN domain-containing metallo enzyme and monocyte chemotactic protein-induced protein might also be responsible for Nanog deubiquitylation.

USP21 has been shown to deubiquitylate and stabilize H2A, RIPK1, GATA3, RIG-1, IL-33 and Tip5.^41–46^ Interestingly, USP21 does not only remove ubiquitin from ubiquitylated proteins but also degrades conjugates of the ubiquitin-like protein ISG15^47^ and according to some reports NEDD8.^48^ In addition, USP21 has an important part in regulating microtubule- and centrosome-associated processes.^49^ Our study identifies the stem cell transcription factor Nanog as a novel substrate of USP21. USP21 binds to the Nanog C-domain, a region that has been demonstrated to have a critical role in transactivation potential.^50^ The Nanog regulator H2A.Z also binds to the C-domain of Nanog and promotes Nanog stability.^51^ Whether H2A.Z cooperates with USP21 to regulate Nanog stability is worthy of further investigation.

Ubiquitin can be conjugated to substrate proteins either as a monoubiquitin or as polyubiquitin chains that varies in length and linkage type. Depending on the lysine residue or the N terminus methionine residue involved in the formation of the polyubiquitin chain, there are a total of eight kinds of ubiquitin linkages that have distinct physiological roles, that is, K6-, K11-, K27-, K29-, K33-, K48-, K63- and M1-linkage (this type is also known as linear ubiquitylation).^39^ K11-, K29- and K48-linked polyubiquitin chains target proteins for proteasome degradation, whereas K63-, M1-linked polyubiquitin chains and multiple monoubiquitin conjugation are preferentially involved in the nondegradative signaling transduction or degradation through the lysosomal pathway.^52^ USP21 can remove the K48- and K63-type of ubiquitin chain depending on the cellular context and the substrate. For instance, USP21 acts as a negative regulator of RIG-I and RIPK1 by removing the K63-linkage of the ubiquitin chain, whereas it deubiquitylates and stabilizes GATA3 by removing the K48-linkage of the ubiquitin chain. Here we found that USP21 prefers to remove K48 linkages of Nanog ubiquitylation and inhibits proteasomal degradation, which is similar to the case of GATA3, another nuclear transcription factor. Furthermore, human Nanog protein contains a total of 18 lysines, most of which are located within the N domain and the central Homeobox domain. Which lysines are ubiquitylated *in vivo* and what is the effect of ubiquitylation on Nanog activity should be explored in future studies.

Nanog is capable of governing the pluripotency of ESCs and early embryonic development.^53,54^ This study shows that USP21 appears to be necessary for the regulation of Nanog. Thus, we propose USP21 may have a role during embryonic development. Depletion of USP21 in mice may lead to abnormalities in development or tissue homeostasis. Recently, two independent studies showed that the *USP21 *knockout mice were viable and fertile, spontaneously developed splenomegaly and were more resistant to vesicular stomatitis virus infection with elevated production of interferons. However, unexpectedly, the knockout mice were not found to have abnormalities in cell morphology, lymphocyte differentiation or hematopoietic stem cell maintenance.^44,55^ To account for this puzzle, we must consider why these phenomena did not occur. First, USP21 may not be the sole ubiquitin-specific protease that catalyzes Nanog deubiquitylation. There might be additional DUBs besides USP21 that work together to regulate Nanog during early embryonic development in mice. Second, USP21 recognizes multiple substrates, either positively or negatively regulating its stability, activity and/or subcellular localization. Therefore, it is not surprising that certain effects might be neutralized or counteracted.

In summary, this study identified USP21 as the specific DUB interacting with Nanog, stabilizing Nanog by deubiquitylating Nanog, and maintaining the pluripotency and self-renewal of mESC. Depletion of USP21 caused mESC differentiation. These findings provide novel insight into the role of USP21 in stem cell fate decision. Future studies are required to determine the physiological functions of USP21–Nanog axis *in vivo*.
